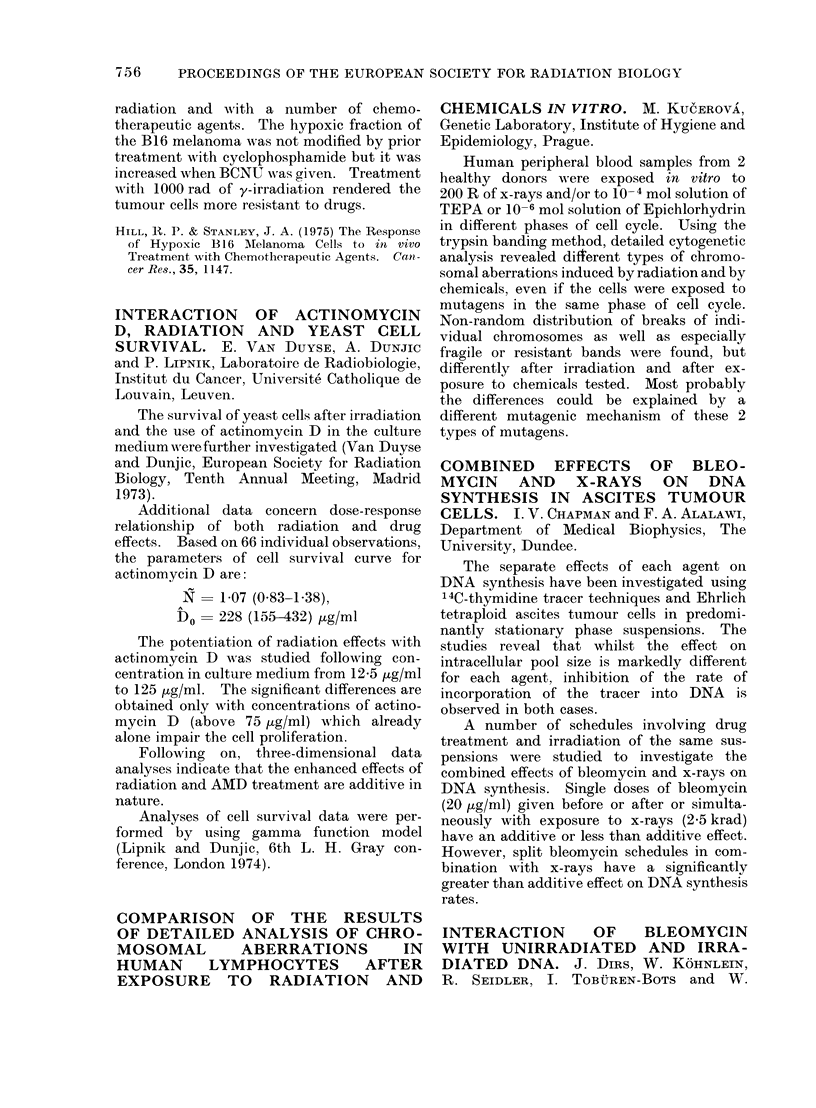# Proceedings: Comparison of the results of detailed analysis of chromosomal aberrations in human lymphocytes after exposure to radiation and chemicals in vitro.

**DOI:** 10.1038/bjc.1975.305

**Published:** 1975-12

**Authors:** M. Kucerová


### COMPARISON OF THE RESULTS OF DETAILED ANALYSIS OF CHROMOSOMAL ABERRATIONS IN HUMAN LYMPHOCYTES AFTER EXPOSURE TO RADIATION AND CHEMICALS *IN VITRO*.

M. KUČEROVÁ, Genetic Laboratory, Institute of Hygiene and Epidemiology, Prague.

Human peripheral blood samples from 2 healthy donors were exposed *in vitro* to 200 R of x-rays and/or to $10^{-4}$ mol solution of TEPA or $10^{-6}$ mol solution of Epichlorhydrin in different phases of cell cycle. Using the trypsin banding method, detailed cytogenetic analysis revealed different types of chromosomal aberrations induced by radiation and by chemicals, even if the cells were exposed to mutagens in the same phase of cell cycle. Non-random distribution of breaks of individual chromosomes as well as especially fragile or resistant bands were found, but differently after irradiation and after exposure to chemicals tested. Most probably the differences could be explained by a different mutagenic mechanism of these 2 types of mutagens.